# 1,1,3,3-Tetra­ethyl­isoindolin-2-ium chloride

**DOI:** 10.1107/S1600536812004588

**Published:** 2012-02-10

**Authors:** Graham Smith, Urs D. Wermuth

**Affiliations:** a Science and Engineering Faculty, Queensland University of Technology, GPO Box 2434, Brisbane, Queensland 4001, Australia

## Abstract

In the title compound, C_16_H_26_N^+^·Cl^−^, the cations and anions form discrete centrosymetric cyclic dimers through N—H⋯Cl hydrogen-bonding associations with graph-set *R*_4_^2^(8).

## Related literature

For the structures of related isoindoline and isoindolinium compounds, see: Fairhurst *et al.* (1996 ▶); Micallef *et al.* (1999 ▶). For the synthesis of alkyl-substituted isoindolines, see: Tönjes *et al.* (1964 ▶); Griffiths *et al.* (1983 ▶). For graph-set anlysis, see: Etter *et al.* (1990 ▶).
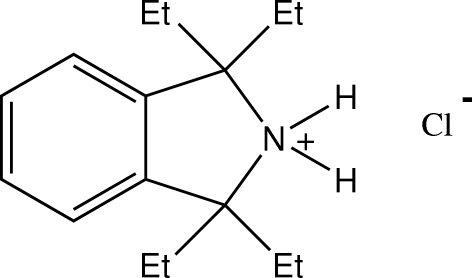


## Experimental

### 

#### Crystal data

C_16_H_26_N^+^·Cl^−^
*M* *_r_* = 267.83
Orthorhombic, 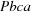

*a* = 12.7282 (4) Å
*b* = 14.0676 (4) Å
*c* = 17.0771 (5) Å
*V* = 3057.74 (16) Å^3^
*Z* = 8
Mo *K*α radiation
μ = 0.24 mm^−1^
*T* = 200 K
0.45 × 0.12 × 0.08 mm

#### Data collection

Oxford Diffraction Gemini-S CCD diffractometer
Absorption correction: multi-scan (*CrysAlis PRO*; Oxford Diffraction, 2010 ▶) *T*_min_ = 0.980, *T*_max_ = 0.990
9765 measured reflections
3007 independent reflections
2126 reflections with *I* > 2σ(*I*)
*R*_int_ = 0.028

#### Refinement

*R*[*F* ^2^ > 2σ(*F* ^2^)] = 0.034
*wR*(*F* ^2^) = 0.086
*S* = 0.91
3007 reflections
171 parameters
H atoms treated by a mixture of independent and constrained refinement
Δρ_max_ = 0.25 e Å^−3^
Δρ_min_ = −0.15 e Å^−3^

### 

Data collection: *CrysAlis PRO* (Oxford Diffraction, 2010 ▶); cell refinement: *CrysAlis PRO*; data reduction: *CrysAlis PRO*; program(s) used to solve structure: *SIR92* (Altomare *et al.*, 1994 ▶); program(s) used to refine structure: *SHELXL97* (Sheldrick, 2008 ▶) within *WinGX* (Farrugia, 1999 ▶); molecular graphics: *PLATON* (Spek, 2009 ▶); software used to prepare material for publication: *PLATON*.

## Supplementary Material

Crystal structure: contains datablock(s) global, I. DOI: 10.1107/S1600536812004588/lh5407sup1.cif

Structure factors: contains datablock(s) I. DOI: 10.1107/S1600536812004588/lh5407Isup2.hkl

Supplementary material file. DOI: 10.1107/S1600536812004588/lh5407Isup3.cml

Additional supplementary materials:  crystallographic information; 3D view; checkCIF report

## Figures and Tables

**Table 1:** Hydrogen-bond geometry (Å, °)

*D*—H⋯*A*	*D*—H	H⋯*A*	*D*⋯*A*	*D*—H⋯*A*
N2—H1*A*⋯Cl1^i^	0.906 (18)	2.322 (18)	3.2054 (15)	165.0 (15)
N2—H1*B*⋯Cl1	0.915 (17)	2.306 (17)	3.1669 (14)	156.8 (14)

Symmetry code: (i) 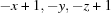.

**Fig. 1.**
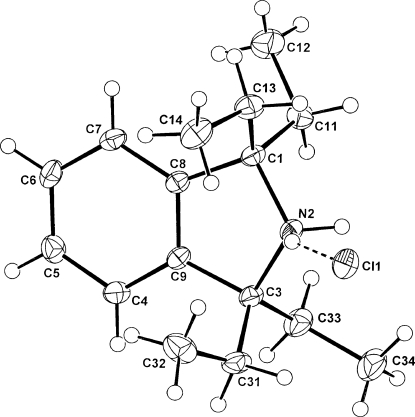
The molecular structure of title compound. The inter-species hydrogen bond is shown as a dashed line and displacement ellipsoids are drawn at the 40% probability level.

**Fig. 2.**
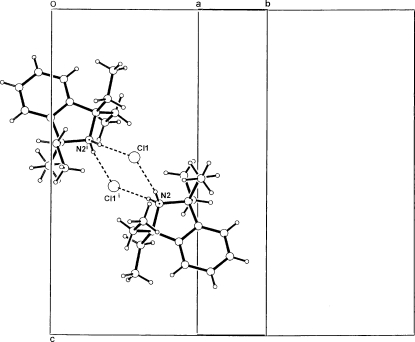
A perspective view of the hydrogen-bonded dimeric structure in the unit cell, showing the centrosymmetric cyclic R24(8) hydrogen-bonding motif. For symmetry code (i), see Table 1.

## supplementary crystallographic information

### Comment

The 1,1,3,3-tetraalkyl-substituted isoindolines have been useful intermediates for the synthesis of nitroxide free-radical scavengers (Griffiths *et al.*, 1983). However, few structures of these compounds are found in the crystallographic literature, *e.g.* 5-nitro-1,1,3,3-tetramethylisoindoline (Fairhurst *et al.*, 1996) and the salt hydrate 1,1,3,3-tetramethylisoindolinium bromide dihydrate (Micallef *et al.*, 1999). The analogous anhydrous 1,1,3,3-tetraethyl-substituted salt C_16_H_26_N^+^ Cl^-^, the title compound, has been synthesized and the structure is reported here.

The molecular structure of the title compound is shown in Fig. 1. The cations and the chloride anions form discrete centrosymetric cyclic dimers through N—H···Cl hydrogen-bonding associations [graph set *R*^2^_4_(8) (Etter *et al.*, 1990)] (Table 1, Fig. 2). The ethyl substituent groups of the molecule adopt various conformations [torsion angles N2—C1—C11—C12, 174.22 (14)°; N2—C1—C13—C14, -69.14 (18)°; N2—C3—C31—C32, 66.66 (17)°; N2—C3—C33—C34, 71.03 (18)°].

### Experimental

The title compound was synthesized using a modification of the method of Tönjes *et al.* (1964) for the synthesis of the 1,1,3,3-tetramethyl analogue (Griffiths *et al.*, 1983). The modification involved the use of ethylmagnesium iodide in the reaction with *N*-benzylphthalimide followed by hydrogenation and conversion to the chloride salt. Colourless needles were obtained from a solution in glacial acetic acid and a specimen was cleaved for the X-ray analysis.

### Refinement

Hydrogen atoms involved in hydrogen-bonding interactions were located in a difference Fourier and their positional and isotropic displacement parameters were refined. Other H-atoms were included in the refinement at calculated positions [C–H = 0.93–0.97 Å, with *U*_iso_(H) = 1.2*U*_eq_ (aromatic or methylene C) or 1.5*U*_eq_(methyl C)], using a riding-model approximation.

### Crystal data

**Table:**

C_16_H_26_N^+^·Cl^−^	*F*(000) = 1168
*M**_r_* = 267.83	*D*_x_ = 1.164 Mg m^−^^3^
Orthorhombic, *P**b**c**a*	Mo *K*α radiation, λ = 0.71073 Å
Hall symbol: -P 2ac 2ab	Cell parameters from 4071 reflections
*a* = 12.7282 (4) Å	θ = 3.2–28.8°
*b* = 14.0676 (4) Å	µ = 0.24 mm^−^^1^
*c* = 17.0771 (5) Å	*T* = 200 K
*V* = 3057.74 (16) Å^3^	Needle, colourless
*Z* = 8	0.45 × 0.12 × 0.08 mm

### Data collection

**Table:**

Oxford Diffraction Gemini-S CCD diffractometer	3007 independent reflections
Radiation source: Enhance (Mo) X-ray source	2126 reflections with *I* > 2σ(*I*)
Graphite monochromator	*R*_int_ = 0.028
Detector resolution: 16.077 pixels mm^-1^	θ_max_ = 26.0°, θ_min_ = 3.2°
ω scans	*h* = −14→15
Absorption correction: multi-scan (*CrysAlis PRO*; Oxford Diffraction, 2010)	*k* = −17→16
*T*_min_ = 0.980, *T*_max_ = 0.990	*l* = −9→21
9765 measured reflections	

### Refinement

**Table:**

Refinement on *F*^2^	Primary atom site location: structure-invariant direct methods
Least-squares matrix: full	Secondary atom site location: difference Fourier map
*R*[*F*^2^ > 2σ(*F*^2^)] = 0.034	Hydrogen site location: inferred from neighbouring sites
*wR*(*F*^2^) = 0.086	H atoms treated by a mixture of independent and constrained refinement
*S* = 0.91	*w* = 1/[σ^2^(*F*_o_^2^) + (0.0507*P*)^2^] where *P* = (*F*_o_^2^ + 2*F*_c_^2^)/3
3007 reflections	(Δ/σ)_max_ < 0.001
171 parameters	Δρ_max_ = 0.25 e Å^−^^3^
0 restraints	Δρ_min_ = −0.15 e Å^−^^3^

### Special details

**Table:**

Geometry. Bond distances, angles *etc*. have been calculated using the rounded fractional coordinates. All su's are estimated from the variances of the (full) variance-covariance matrix. The cell e.s.d.'s are taken into account in the estimation of distances, angles and torsion angles
Refinement. Refinement of *F*^2^ against ALL reflections. The weighted *R*-factor *wR* and goodness of fit *S* are based on *F*^2^, conventional *R*-factors *R* are based on *F*, with *F* set to zero for negative *F*^2^. The threshold expression of *F*^2^ > σ(*F*^2^) is used only for calculating *R*-factors(gt) *etc*. and is not relevant to the choice of reflections for refinement. *R*-factors based on *F*^2^ are statistically about twice as large as those based on *F*, and *R*- factors based on ALL data will be even larger.

### Fractional atomic coordinates and isotropic or equivalent isotropic displacement parameters (Å^2^)

**Table:**

	*x*	*y*	*z*	*U*_iso_*/*U*_eq_	
N2	0.57440 (10)	0.11256 (10)	0.59705 (8)	0.0215 (4)	
C1	0.53181 (12)	0.11091 (11)	0.68132 (9)	0.0231 (5)	
C3	0.66601 (12)	0.18396 (10)	0.58973 (9)	0.0228 (5)	
C4	0.71046 (12)	0.31935 (11)	0.68650 (9)	0.0265 (5)	
C5	0.68541 (13)	0.36369 (11)	0.75667 (10)	0.0294 (5)	
C6	0.60529 (13)	0.32973 (11)	0.80353 (9)	0.0281 (5)	
C7	0.55023 (12)	0.24882 (11)	0.78244 (9)	0.0262 (5)	
C8	0.57612 (12)	0.20269 (10)	0.71300 (9)	0.0213 (5)	
C9	0.65433 (11)	0.23876 (10)	0.66514 (9)	0.0210 (5)	
C11	0.58119 (13)	0.02388 (11)	0.72323 (9)	0.0296 (5)	
C12	0.55776 (16)	0.01439 (14)	0.81002 (10)	0.0443 (7)	
C13	0.41208 (12)	0.10409 (12)	0.68007 (10)	0.0306 (5)	
C14	0.35314 (13)	0.19029 (12)	0.65082 (11)	0.0391 (6)	
C31	0.65211 (13)	0.24731 (12)	0.51751 (9)	0.0313 (6)	
C32	0.55947 (15)	0.31571 (13)	0.51940 (11)	0.0406 (6)	
C33	0.77174 (12)	0.13089 (12)	0.58608 (11)	0.0338 (6)	
C34	0.79401 (15)	0.07602 (14)	0.51062 (12)	0.0484 (7)	
Cl1	0.41410 (3)	0.10600 (3)	0.45532 (2)	0.0300 (1)	
H1A	0.5909 (13)	0.0523 (13)	0.5829 (11)	0.038 (5)*	
H1B	0.5243 (13)	0.1283 (11)	0.5609 (10)	0.025 (4)*	
H4	0.76350	0.34300	0.65460	0.0320*	
H5	0.72310	0.41700	0.77240	0.0350*	
H6	0.58820	0.36150	0.84960	0.0340*	
H7	0.49670	0.22560	0.81420	0.0310*	
H11A	0.55690	−0.03330	0.69710	0.0350*	
H11B	0.65680	0.02690	0.71660	0.0350*	
H12A	0.59160	−0.04150	0.83010	0.0660*	
H12B	0.48330	0.00930	0.81770	0.0660*	
H12C	0.58360	0.06940	0.83720	0.0660*	
H13A	0.39270	0.05030	0.64760	0.0370*	
H13B	0.38830	0.09050	0.73280	0.0370*	
H14A	0.27900	0.17790	0.65230	0.0590*	
H14B	0.37400	0.20390	0.59800	0.0590*	
H14C	0.36890	0.24390	0.68360	0.0590*	
H31A	0.71590	0.28410	0.51050	0.0370*	
H31B	0.64460	0.20660	0.47200	0.0370*	
H32A	0.55800	0.35200	0.47180	0.0610*	
H32B	0.56670	0.35790	0.56320	0.0610*	
H32C	0.49530	0.28030	0.52440	0.0610*	
H33A	0.82770	0.17680	0.59370	0.0410*	
H33B	0.77460	0.08650	0.62950	0.0410*	
H34A	0.86140	0.04570	0.51440	0.0730*	
H34B	0.79410	0.11920	0.46710	0.0730*	
H34C	0.74060	0.02880	0.50300	0.0730*	

### Atomic displacement parameters (Å^2^)

**Table:**

	*U*^11^	*U*^22^	*U*^33^	*U*^12^	*U*^13^	*U*^23^
N2	0.0211 (7)	0.0257 (7)	0.0177 (7)	−0.0002 (6)	−0.0007 (6)	−0.0027 (6)
C1	0.0233 (8)	0.0279 (8)	0.0181 (8)	−0.0014 (7)	0.0032 (6)	−0.0008 (7)
C3	0.0205 (8)	0.0284 (8)	0.0196 (8)	−0.0049 (7)	0.0027 (6)	−0.0033 (7)
C4	0.0260 (9)	0.0310 (9)	0.0226 (9)	−0.0042 (7)	0.0025 (7)	0.0020 (7)
C5	0.0354 (10)	0.0260 (8)	0.0269 (9)	−0.0036 (7)	−0.0052 (7)	−0.0031 (7)
C6	0.0367 (10)	0.0283 (9)	0.0194 (8)	0.0081 (7)	−0.0007 (7)	−0.0042 (7)
C7	0.0277 (9)	0.0314 (9)	0.0195 (8)	0.0033 (7)	0.0047 (6)	0.0037 (7)
C8	0.0206 (8)	0.0250 (8)	0.0182 (8)	0.0022 (6)	−0.0002 (6)	0.0010 (7)
C9	0.0195 (8)	0.0262 (8)	0.0173 (8)	0.0028 (7)	−0.0007 (6)	−0.0006 (7)
C11	0.0345 (10)	0.0274 (9)	0.0269 (9)	0.0008 (8)	−0.0001 (8)	0.0027 (7)
C12	0.0594 (13)	0.0461 (11)	0.0274 (10)	0.0057 (10)	0.0009 (9)	0.0078 (9)
C13	0.0241 (8)	0.0378 (9)	0.0298 (9)	−0.0025 (8)	0.0038 (7)	0.0009 (8)
C14	0.0277 (10)	0.0511 (11)	0.0384 (11)	0.0086 (9)	0.0028 (8)	−0.0024 (9)
C31	0.0369 (10)	0.0400 (10)	0.0169 (9)	−0.0107 (8)	0.0024 (7)	−0.0002 (8)
C32	0.0508 (12)	0.0372 (10)	0.0338 (10)	−0.0044 (9)	−0.0092 (8)	0.0118 (9)
C33	0.0225 (9)	0.0419 (10)	0.0370 (11)	−0.0001 (8)	0.0066 (7)	−0.0069 (8)
C34	0.0368 (11)	0.0508 (12)	0.0576 (14)	−0.0053 (9)	0.0198 (10)	−0.0192 (11)
Cl1	0.0298 (2)	0.0341 (2)	0.0260 (2)	0.0029 (2)	−0.0059 (2)	−0.0047 (2)

### Geometric parameters (Å, º)

**Table:**

N2—C1	1.538 (2)	C6—H6	0.9300
N2—C3	1.544 (2)	C7—H7	0.9300
N2—H1A	0.906 (18)	C11—H11A	0.9700
N2—H1B	0.915 (17)	C11—H11B	0.9700
C1—C8	1.509 (2)	C12—H12A	0.9600
C1—C13	1.527 (2)	C12—H12B	0.9600
C1—C11	1.551 (2)	C12—H12C	0.9600
C3—C31	1.532 (2)	C13—H13A	0.9700
C3—C33	1.540 (2)	C13—H13B	0.9700
C3—C9	1.508 (2)	C14—H14A	0.9600
C4—C5	1.388 (2)	C14—H14B	0.9600
C4—C9	1.389 (2)	C14—H14C	0.9600
C5—C6	1.382 (2)	C31—H31A	0.9700
C6—C7	1.384 (2)	C31—H31B	0.9700
C7—C8	1.391 (2)	C32—H32A	0.9600
C8—C9	1.384 (2)	C32—H32B	0.9600
C11—C12	1.518 (2)	C32—H32C	0.9600
C13—C14	1.511 (2)	C33—H33A	0.9700
C31—C32	1.522 (3)	C33—H33B	0.9700
C33—C34	1.529 (3)	C34—H34A	0.9600
C4—H4	0.9300	C34—H34B	0.9600
C5—H5	0.9300	C34—H34C	0.9600
			
C1—N2—C3	110.59 (12)	C12—C11—H11A	108.00
H1A—N2—H1B	102.0 (15)	C12—C11—H11B	108.00
C1—N2—H1A	108.5 (12)	H11A—C11—H11B	107.00
C1—N2—H1B	112.9 (11)	C11—C12—H12A	109.00
C3—N2—H1A	114.3 (11)	C11—C12—H12B	110.00
C3—N2—H1B	108.3 (10)	C11—C12—H12C	109.00
N2—C1—C13	109.85 (12)	H12A—C12—H12B	109.00
C8—C1—C11	111.00 (12)	H12A—C12—H12C	109.00
N2—C1—C11	107.51 (12)	H12B—C12—H12C	109.00
N2—C1—C8	101.00 (12)	C1—C13—H13A	108.00
C11—C1—C13	111.17 (13)	C1—C13—H13B	108.00
C8—C1—C13	115.57 (13)	C14—C13—H13A	108.00
C9—C3—C33	111.62 (13)	C14—C13—H13B	108.00
C9—C3—C31	112.25 (12)	H13A—C13—H13B	107.00
N2—C3—C9	100.89 (12)	C13—C14—H14A	109.00
N2—C3—C31	110.88 (12)	C13—C14—H14B	109.00
N2—C3—C33	110.35 (12)	C13—C14—H14C	110.00
C31—C3—C33	110.51 (13)	H14A—C14—H14B	109.00
C5—C4—C9	118.39 (14)	H14A—C14—H14C	109.00
C4—C5—C6	120.95 (15)	H14B—C14—H14C	109.00
C5—C6—C7	120.49 (14)	C3—C31—H31A	108.00
C6—C7—C8	119.03 (14)	C3—C31—H31B	108.00
C1—C8—C9	111.76 (13)	C32—C31—H31A	108.00
C7—C8—C9	120.17 (14)	C32—C31—H31B	108.00
C1—C8—C7	128.03 (14)	H31A—C31—H31B	107.00
C3—C9—C8	112.81 (13)	C31—C32—H32A	109.00
C4—C9—C8	120.94 (14)	C31—C32—H32B	109.00
C3—C9—C4	126.21 (13)	C31—C32—H32C	109.00
C1—C11—C12	116.13 (14)	H32A—C32—H32B	109.00
C1—C13—C14	116.72 (14)	H32A—C32—H32C	110.00
C3—C31—C32	116.11 (14)	H32B—C32—H32C	109.00
C3—C33—C34	116.16 (14)	C3—C33—H33A	108.00
C5—C4—H4	121.00	C3—C33—H33B	108.00
C9—C4—H4	121.00	C34—C33—H33A	108.00
C4—C5—H5	120.00	C34—C33—H33B	108.00
C6—C5—H5	119.00	H33A—C33—H33B	107.00
C5—C6—H6	120.00	C33—C34—H34A	109.00
C7—C6—H6	120.00	C33—C34—H34B	109.00
C6—C7—H7	121.00	C33—C34—H34C	109.00
C8—C7—H7	120.00	H34A—C34—H34B	109.00
C1—C11—H11A	108.00	H34A—C34—H34C	110.00
C1—C11—H11B	108.00	H34B—C34—H34C	110.00
			
C3—N2—C1—C8	17.41 (15)	C31—C3—C9—C8	122.53 (14)
C3—N2—C1—C11	−98.96 (14)	C33—C3—C9—C4	69.65 (19)
C3—N2—C1—C13	139.94 (13)	C33—C3—C9—C8	−112.77 (15)
C1—N2—C3—C33	104.26 (14)	N2—C3—C31—C32	66.66 (17)
C1—N2—C3—C9	−13.87 (15)	C9—C3—C31—C32	−45.33 (19)
C1—N2—C3—C31	−132.96 (13)	C33—C3—C31—C32	−170.65 (14)
N2—C1—C8—C9	−14.63 (16)	N2—C3—C33—C34	71.03 (18)
C13—C1—C8—C7	49.6 (2)	C9—C3—C33—C34	−177.64 (14)
C11—C1—C8—C7	−78.24 (19)	C31—C3—C33—C34	−51.97 (18)
C11—C1—C8—C9	99.12 (15)	C9—C4—C5—C6	−1.2 (2)
N2—C1—C8—C7	168.01 (15)	C5—C4—C9—C3	176.62 (14)
C13—C1—C11—C12	−65.51 (18)	C5—C4—C9—C8	−0.8 (2)
N2—C1—C13—C14	−69.14 (18)	C4—C5—C6—C7	1.9 (2)
C8—C1—C13—C14	44.3 (2)	C5—C6—C7—C8	−0.6 (2)
C11—C1—C13—C14	171.99 (14)	C6—C7—C8—C1	175.77 (15)
C13—C1—C8—C9	−133.09 (14)	C6—C7—C8—C9	−1.4 (2)
N2—C1—C11—C12	174.22 (14)	C1—C8—C9—C3	6.78 (18)
C8—C1—C11—C12	64.62 (18)	C1—C8—C9—C4	−175.50 (14)
N2—C3—C9—C4	−173.14 (14)	C7—C8—C9—C3	−175.63 (13)
N2—C3—C9—C8	4.44 (16)	C7—C8—C9—C4	2.1 (2)
C31—C3—C9—C4	−55.1 (2)		

### Hydrogen-bond geometry (Å, º)

**Table:**

*D*—H···*A*	*D*—H	H···*A*	*D*···*A*	*D*—H···*A*
N2—H1*A*···Cl1^i^	0.906 (18)	2.322 (18)	3.2054 (15)	165.0 (15)
N2—H1*B*···Cl1	0.915 (17)	2.306 (17)	3.1669 (14)	156.8 (14)
C4—H4···Cl1^ii^	0.93	2.78	3.6995 (16)	172
C11—H11*A*···Cl1^i^	0.97	2.82	3.5551 (16)	133
C34—H34*C*···Cl1^i^	0.96	2.82	3.730 (2)	158

Symmetry codes: (i) −*x*+1, −*y*, −*z*+1; (ii) *x*+1/2, −*y*+1/2, −*z*+1.

## References

[bb1] Altomare, A., Cascarano, G., Giacovazzo, C., Guagliardi, A., Burla, M. C., Polidori, G. & Camalli, M. (1994). *J. Appl. Cryst.* **27**, 435.

[bb2] Etter, M. C., MacDonald, J. C. & Bernstein, J. (1990). *Acta Cryst.* B**46**, 256–262. 10.1107/s0108768189012929 2344397

[bb3] Fairhurst, S. A., Gillies, D. G., Smith, G. W., Sutcliffe, L. H. & Wu, X. (1996). *J. Mol. Struct.* **375**, 105–115.

[bb4] Farrugia, L. J. (1999). *J. Appl. Cryst.* **32**, 837–838.

[bb5] Griffiths, P. G., Moad, G., Rizzardo, E. & Solomon, D. H. (1983). *Aust. J. Chem.* **36**, 397–401.

[bb6] Micallef, A. S., Bott, R. C., Bottle, S. E., Smith, G., White, J. M., Matsuda, K. & Iwamura, H. (1999). *J. Chem. Soc. Perkin Trans. 2*, pp. 65–71.

[bb7] Oxford Diffraction (2010). *CrysAlis PRO* Oxford Diffraction Ltd, Yarnton, Oxfordshire, England.

[bb8] Sheldrick, G. M. (2008). *Acta Cryst.* A**64**, 112–122. 10.1107/S0108767307043930 18156677

[bb9] Spek, A. L. (2009). *Acta Cryst.* D**65**, 148–155. 10.1107/S090744490804362X PMC2631630 19171970

[bb10] Tönjes, H., Heidenbluth, K. & Scheffler, R. (1964). *J. Prakt. Chem.* **26**, 218–224.

